# Important marine areas for endangered African penguins before and after the crucial stage of moulting

**DOI:** 10.1038/s41598-022-12969-w

**Published:** 2022-06-08

**Authors:** Tegan Carpenter-Kling, Andrew de Blocq, Christina Hagen, Craig Harding, Taryn Morris, Lorien Pichegru, Jennifer Roberts, Peter G. Ryan, Ross M. Wanless, Alistair McInnes

**Affiliations:** 1 BirdLife South Africa, Seabird Conservation Programme, Cape Town, 8001 South Africa; 2 Institute for Coastal and Marine Research and Department of Zoology, Nelson Mandela University, Port Elizabeth, 6001 South Africa; 3 FitzPatrick Institute of African Ornithology, University of Cape Town, Cape Town, 7700 South Africa

**Keywords:** Animal behaviour, Animal migration, Conservation biology

## Abstract

The population of the Endangered African penguin *Spheniscus demersus* has decreased by > 65% in the last 20 years. A major driver of this decrease has been the reduced availability of their principal prey, sardine *Sardinops sagax* and anchovy *Engraulis encrasicolus.* To date, conservation efforts to improve prey availability have focused on spatial management strategies to reduce resource competition with purse-seine fisheries during the breeding season. However, penguins also undergo an annual catastrophic moult when they are unable to feed for several weeks. Before moulting they must accumulate sufficient energy stores to survive this critical life-history stage. Using GPS tracking data collected between 2012 and 2019, we identify important foraging areas for pre- and post-moult African penguins at three of their major colonies in South Africa: Dassen Island and Stony Point (Western Cape) and Bird Island (Eastern Cape). The foraging ranges of pre- and post-moult adult African penguins (c. 600 km from colony) was far greater than that previously observed for breeding penguins (c. 50 km from colony) and varied considerably between sites, years and pre- and post-moult stages. Despite their more extensive range during the non-breeding season, waters within 20 and 50 km of their breeding colonies were used intensively and represent important foraging areas to pre- and post-moult penguins. Furthermore, penguins in the Western Cape travelled significantly further than those in the Eastern Cape which is likely a reflection of the poor prey availability along the west coast of South Africa. Our findings identify important marine areas for pre- and post-moult African penguins and support for the expansion of fisheries-related spatio-temporal management strategies to help conserve African penguins outside the breeding season.

## Introduction

Marine ecosystems are under severe pressure from anthropogenic activities^1^, with negative impacts being observed throughout the trophic web^2^. Since 1950, the size of seabirds’ populations are estimated to have decreased by c. 70%^3^ and consequently, seabirds are one of the most threatened groups of birds globally^4,5^. They face threats on land and at sea, such as resource depletion due to climate change or competition with fisheries, introduced predators and diseases, breeding habitat loss, and mortality linked to fisheries bycatch^5–8^. No-take zones and Marine Protected Areas (MPAs) have been proposed as the most appropriate strategies to mitigate threats associated with prey availability^2,7,9^. However, in-depth knowledge of how a species uses its environment during different life-history stages is required at appropriate spatio-temporal scales to inform conservation strategies^10–12^.

While breeding, the foraging range of seabirds is constrained around a central location, as they must return regularly to incubate eggs and care for dependent young. These foraging constraints are relaxed outside of the breeding season, when seabirds often extend their distribution and target foraging habitats outside of their breeding foraging range^13,14^. The larger distribution of non-breeding seabirds and their increased amount of time spent at sea increases the probability of the birds interacting with potentially risky anthropogenic activities^15,16^. Thus, many studies focus primarily on bycatch risk when investigating relationships between fisheries and non-breeding seabirds^16–18^. Nonetheless, distributional overlap of non-breeding seabirds and fisheries indicates there is a potential for seabird-fisheries competition for resources outside of the breeding season^19,20^. Previously environmentally mediated variability in prey availability has been shown to influence the non-breeding distributions of seabirds and which have led to carry-over effects into the proceeding breeding season^21–26^. Therefore, if fisheries influence the availability of the seabirds’ prey outside the breeding season it will likely influence non-breeding seabirds’ foraging behaviour, in a similar way as fisheries influence the foraging behaviour and breeding success of breeding seabirds^27–29^.

The post-breeding and pre-moult periods are energetically demanding for seabirds as they undergo plumage replacement and restore energy reserves lost during the breeding season^30,31^. For penguins, the energy demands of this period are particularly intense as, unlike other seabirds which stagger their moult, penguins replace their entire plumage in 2–5 weeks, in a so-called catastrophic moult^32^. While moulting, penguins are land-bound and are entirely dependent on endogenous reserves^33^, resulting in a 40–50% loss in body mass over the moult period^34^. Penguins starve if they do not commence their moult with sufficient fat reserves to complete the moult and return to sea^33,34^. Consequently, penguin moult is often marked by higher adult mortality^35^. And even if adult penguins survive the moult, poor foraging conditions during the post-moult recovery period can have carry-over effects into the following breeding season^36–38^. These factors could have serious negative consequences on individual fitness and associated population growth, highlighting the importance of the non-breeding period for penguin conservation strategies.

Poor forage fish availability since the late 1990s along the west coast of South Africa has coincided with population declines of three endemic seabirds that predominantly prey on *Sardinops sagax* and anchovy *Engraulis encrasicolus*: African penguins, Cape gannets *Morus Capensis* and Cape cormorants *Phalacrocorax capensis*^39^. The declines have been attributed to insufficient availability of high quality prey or increased foraging effort to reach profitable foraging areas, resulting in lower reproductive success and survival^40–44^. However, despite the biomass of sardine along the west coast consistently being below a critical threshold for these birds’ survival during the past decade^23,45^, fishing for sardine has continued.

The African penguin *Spheniscus demersus* was listed as Endangered in 2010^46^, following a > 65% population decrease since 1989^47^. This trend has largely been attributed to the reduced availability of their preferred prey^23,39,46,48^ with pollution (oil spills), habitat destruction, human disturbance, disease, and predation also contributing to losses^46,49,50^. The purse-seine fishery is the largest extractor of fish biomass in South Africa which mostly targets sardine and anchovy. There is considerable overlap with the distributions of purse-seine catches and foraging African Penguins during the breeding season^51^. The influence of resource competition by the purse-seine fishery on the demographic parameters of breeding African penguins has been investigated in an experiment that included two paired breeding island groups with alternating closures every 3 years^24,52^. The results have shown positive impacts of no-take zones on breeding and foraging parameters^24,52,53^, although these results have been contested^54,55^. However, the design of these no-take zones (i.e. 20 km radius) were informed by the foraging range of breeding African penguins (< 50 km)^41,56^. Therefore they do not consider the much greater distribution of the African penguin outside of the breeding season^57^, despite the importance of sardine biomass during the pre- and post-moult stages on subsequent breeding effort and adult survival^23,24^.

Here, using tracking data collected between 2012 and 2019, we examine the pre- and post- moult foraging distribution of African penguins from three of their largest colonies, Bird Island, Dassen Island and Stony Point, which in 2019 had 1912, 1705 and 2378 breeding pairs, respectively, which relates to 11.1%, 9.9% and 13.8% of the global African penguin population^47^. Inter-annual and inter-stage differences in the foraging distributions are assessed and we use tracking data to define pre- and post-moult Important Bird Areas (IBA) for each colony using standardised methods developed by BirdLife International^58,59^. The IBA’s will be used to examine the overlap of these areas with potential threats, including resource competition, in an effort to understand where potential management interventions, e.g. spatio-temporal management of fishing fleets, can be most effectively applied. These layers will also be submitted as biodiversity feature layers to inform various marine spatial planning initiatives including the expansion of Marine Protected Areas in South Africa^60–62^.

## Results

Over the study period, 81 pre-moult and 19 post-moult African penguin foraging trips lasting > 20 days were recorded from Bird Island (19 and 6 individuals, respectively; 2012–2015) and Dassen Island (50 and 13 individuals, respectively; 2012–2019) and Stony Point (12 pre-moult individuals; 2018–2019 Table 1). Distributions were significantly different between stages, colonies and years, with penguins from Dassen Island travelling the farthest during both the pre- and post-moult stages compared to penguins from Bird Island and Stony Point.

**Table 1:** The number of African penguins tracked for > 20 days from Dassen Island, Stony Point and Bird Island during their pre- and post-moult foraging trips between 2012 and 2019.

Year	Device type	Number of individuals (first deployment date)	Mean ± STD of number of days tracked	Length and (number) of complete pre- moult trips tracked	Number of individuals (first deployment date)	Number of days tracked (mean ± STD)
		Pre-moult			Post-moult	
**Bird Island**						
2012	PTT^a^	10 (13 Sep)	51.7 ± 14.4			
2013	PTT^a^	6 (12 Sep)	80.0 ± 5.7		5 (6 Dec)	45.0 ± 8.3
2014	Catlog^b^				1 (23 Nov)	45
2015	Catlog^b^	3 (24 Sep)	60.7 ± 3.2			
Total number of birds tracked:		19			6	
**Dassen Island**						
2012	PTT^a^	8 (11 Sep)	50.5 ± 22.5			
2013	PTT^a^	10 (5 Sep)	46.8 ± 18.8		4 (16 Nov)	67.3 ± 18.2
2014	Catlog^b^/PTT^b^	6 (17 Aug)	60.3 ± 38.2		3 (7 Nov)	58.3 ± 10.7
2015	Catlog^b^/PTT^b^	4 (15 Sep)	44.5 ± 21.1		3 (7 Nov)	72.3 ± 31.5
2017	GPS-GSM^c^	4 (23 Sep)	29.3 ± 4.7	27.0 ± 4.6 (4)		
2018	GPS-GSM^c^	10 (31 Aug)	34.7 ± 6.6	35.3 ± 6.9 (7)	2 (18 Oct)	37.5 ± 20.5
2019	GPS-GSM	8 (12 Sep)	40.0 ± 9.9	42.7 ± 7.7 (6)	1 (10 Nov)	58
Total number of birds tracked:		50			13	
**Stony point**						
2018	GPS-GSM^c^	10 (11 Oct)	35.1 ± 10.4	31.6 ± 5.9 (9)		
2019	GPS-GSM^c^	2 (17 Oct)	27 & 29	27 &29 (2)		
Total number of birds tracked:		12				

^a^KiwiSat202, SirTrack, 58 × 28 × 18 mm with 180 mm antennae, 40 g.

^b^CatLog-S, Perthold Engineering LLC USA, 50 × 22 × 8 mm, 34 g.

^c^Pathtrack Limited, 63 × 20 × 18 mm with 40 mm antennae, 25 g.

### Pre- and post-moult distribution of African penguins

**Figure 1:**
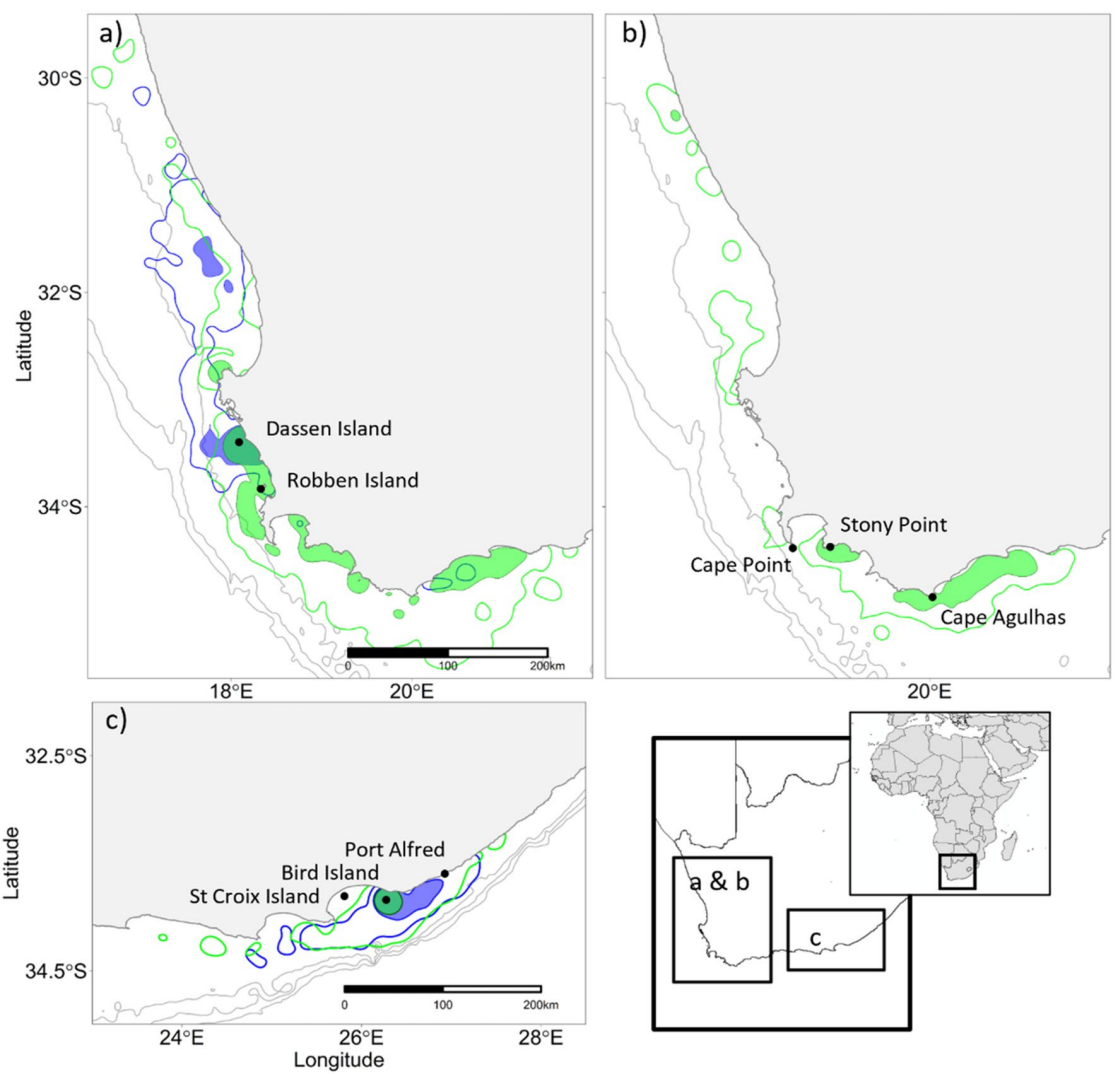
The distributional range (90% utilisation distribution—UD, open polygon) and core range (54% UD, shaded area) of African penguins tagged at (**a**) Dassen Island, (**b**) Stony Point, and (**c**) Bird Island during their pre- (green) and post-(blue) moult foraging trips to the 200, 500 and 1000 m isobaths (grey lines).

**Figure 2:**
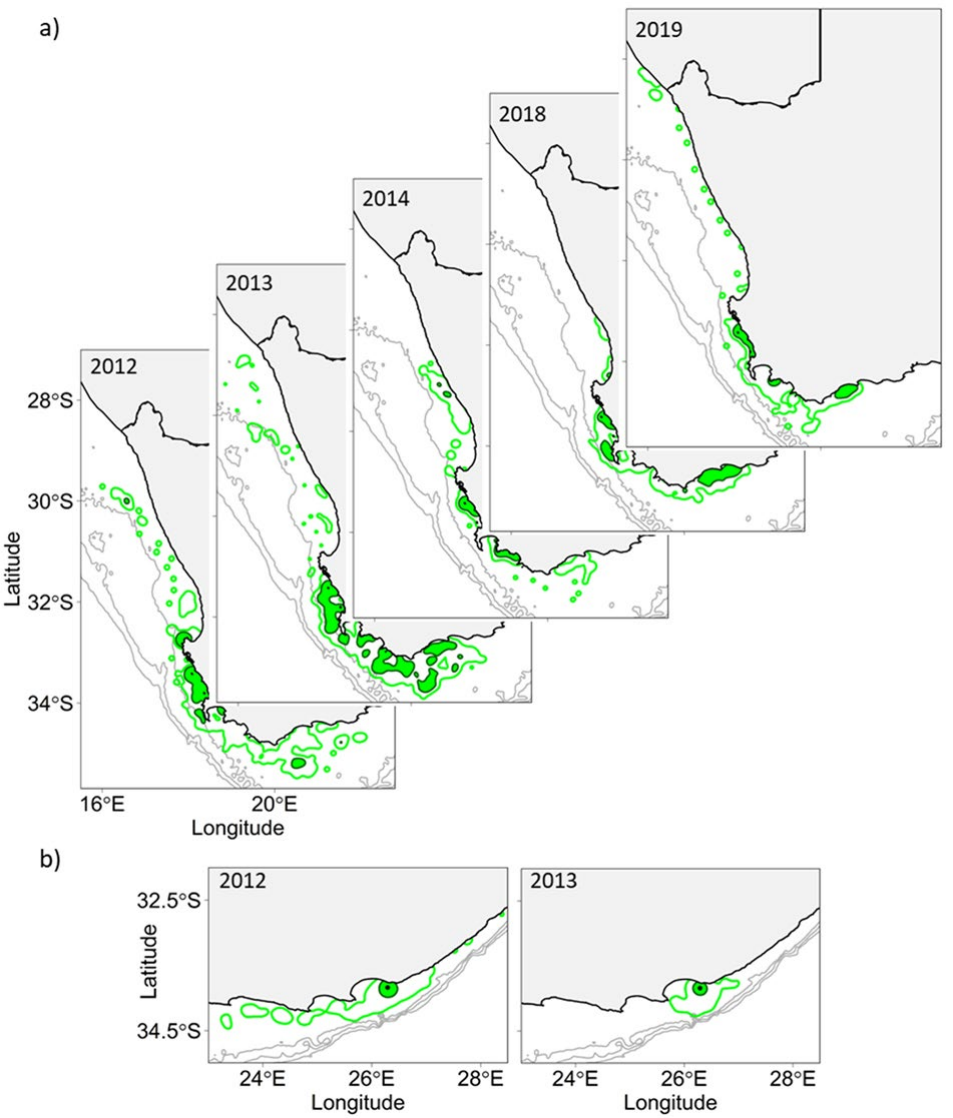
Annual distributional (90% utilisation distribution—UD, open polygon) and core ranges (54% UD, shaded area) of African penguins tagged at (**a**) Dassen Island and (**b**) Bird Island during their pre-moult foraging trips to the 200, 500 and 1000 m isobaths (grey lines). Annual distributional and core ranges were only calculated when > 5 individuals were tracked.

During pre-moult trips, penguins from both Dassen Island and Stony Point mostly travelled south-eastward, with core ranges within the vicinity of their colonies and east of Cape Agulhas. In contrast, post-moult birds from Dassen Island mostly travelled north of St Helena Bay (Fig. 1). Pre- and post-moult birds from Bird Island remained close to the colony, but the post-moult core range was larger than the pre-moult range (Fig. 1). Permutation tests revealed that pre-and post-moult distributions were significantly different at both the core (54% UD) and distributional ranges (90% UD) of the Bird Island and Dassen Island penguins (Table 2, Fig. 1). In addition, there was significant interannual variability in the distribution of Dassen Island and Bird Island pre-moult penguins as shown by the permutation test (Fig. 2; Table 2).

**Table 2:** Utilisation distribution (UD) overlap (Bhattacharyya’s Affinity^119^) of pre- and post-moult African penguins at the core (54% UD) and distributional range (90% UD) between (a) pre- and post-moult stages, (b) year and stage at Bird Island, and (c) year and stage at Dassen Island.

	At the 54% UD			At the 90% UD		
	Observed overlap	Permuted overlap (mean ± SD)	p-value (95% CI)	Observed overlap	Permuted overlap (mean ± SD)	p-value (95% CI)
**(a) Stage comparisons (pre-moult vs. post-moult)**						
Bird Island	42.0	50.5 ± 2.4	** < 0.01 (< 0.01–0.01)**	80.4	82.7 ± 1.8	0.10 (0.08–0.11)
Dassen Island	29.6	37.1 ± 3.01	**0.01 (0.01–0.02)**	44.0	68.3 ± 3.3	** < 0.01 (< 0.01**–**< 0.01)**
**(b) Year comparison of Bird Island pre-moult UDs**						
2012 vs 2013	47.8	51.6 ± 1.5	**0.02 (0.01–0.03)**	81.0	83.3 ± 1.2	**0.05 (0.04**–**0.06)**
**(c) Year comparison of Dassen Island pre-moult UDs**						
2012 vs 2013	21.4	22.67 ± 2.1	0.25 (0.22–0.28)	56.0	55.8 ± 2.9	0.49 (0.46–0.52)
2012 vs 2014	27.4	26.7 ± 2.9	0.53 (0.50–0.56)	48.1	47.8 ± 4.4	0.52 (0.49–0.55)
2012 vs 2018	18.9	27.5 ± 3.3	**0.01 (< 0.01–0.01)**	50.5	56.6 ± 4.2	0.08 (0.07–0.1)
2012 vs 2019	24.0	30.4 ± 3.45	**0.05 (0.03–0.06)**	51.1	55.1 ± 3.7	0.13 (0.11–0.16)
2013 vs 2014	21.7	21.7 ± 3.7	0.48 (0.45–0.51)	38.7	53.0 ± 5.3	**0.01 (< 0.01**–**0.02)**
2013 vs 2018	21.6	28.2 ± 3.8	0.06 (0.05–0.08)	54.9	64.7 ± 3.6	**0.02 (0.01**–**0.03)**
2013 vs 2019	22.6	28.0 ± 3.4	0.07 (0.06–0.09)	53.5	62.4 ± 4.1	**0.03 (0.02**–**0.04)**
2014 vs 2018	25.7	26.8 ± 4.7	0.36 (0.33–0.39)	49.1	56.34 ± 6.8	0.13 (0.11–0.15)
2014 vs 2019	34.5	32.4 ± 5.7	0.63 (0.60–0.66)	56.6	59.5 ± 7.3	0.34 (0.31–0.37)
2018 vs 2019	35.7	35.5 ± 4.0	0.44 (0.41–0.47)	65.8	67.3 ± 4.3	0.29 (0.26–0.32)

Significantly different UDs (bold) were identified by comparing the real (observed) overlap to the distribution of overlaps from 1000 permutations of either the stage or year labels where appropriate.

Pre-moult penguins dispersing from Stony Point were found to return to Stony Point to perform their annual moult. In contrast, 50% of the pre-moult penguins dispersing from Dassen Island, for which moulting could be determine, moulted at Dassen Island and 50% moulted at Stony Point. However, the location of moult for penguins could only be determined for penguins which were tracked with GPS-GSMs tracking devices (see “Methods” for more detail).

**Figure 3:**
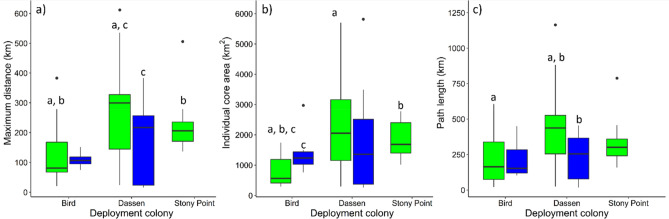
Box plots (median, interquartile (IQR_3_ and IQR_4_) range, minimum (IQR_3_ * 1.5) and maximum (IQR_4_) values and outliers) showing inter-site comparisons of African penguin path metrics during pre-moult (green) and post-moult (blue) stages: (**a**) maximum distance travelled from deployment colony (**b**) area of individual core areas (i.e. 54% utilisation distribution—UD), and (**c**) path length of maximum distance travelled from deployment colony. Letters indicate a p-value of < 0. 05 between colony or stages comparisons investigated using a PERNOVA or Dunn’s test were appropriate.

The pre-moult path metrics were significantly different between colonies (perANOVA: p < 0.01 for all path metrics), with penguins from Dassen Island and Stony Point travelling significantly further than penguins from Bird Island (Fig. 3; Supplementary Table S1). Path metrics were also significantly different between pre- and post-moult penguins from Dassen Island (perANOVAs: path length: p = 0.01; maximum distance: p = 0.03; individual core areas: p = 0.28) and Bird Island (perANOVAs: path length: p = 0.98; maximum distance: p = 0.68; core areas: p = 0.02; Fig. 3). Pre-moult penguins from Dassen Island travelled significantly longer distances (path length) and significantly further from their colonies (maximum distance) than post-moult penguins from Dassen Island (Fig. 3; Supplementary Table S1). In contrast, for penguins from Bird Island, path lengths and maximum distances were similar between pre- and post-moult trips, whereas individual core areas were significantly greater for post-moulters compared to pre-moulters (Fig. 3; Supplementary Table S1).

### Identification of marine Important Bird Areas

**Figure 4:**
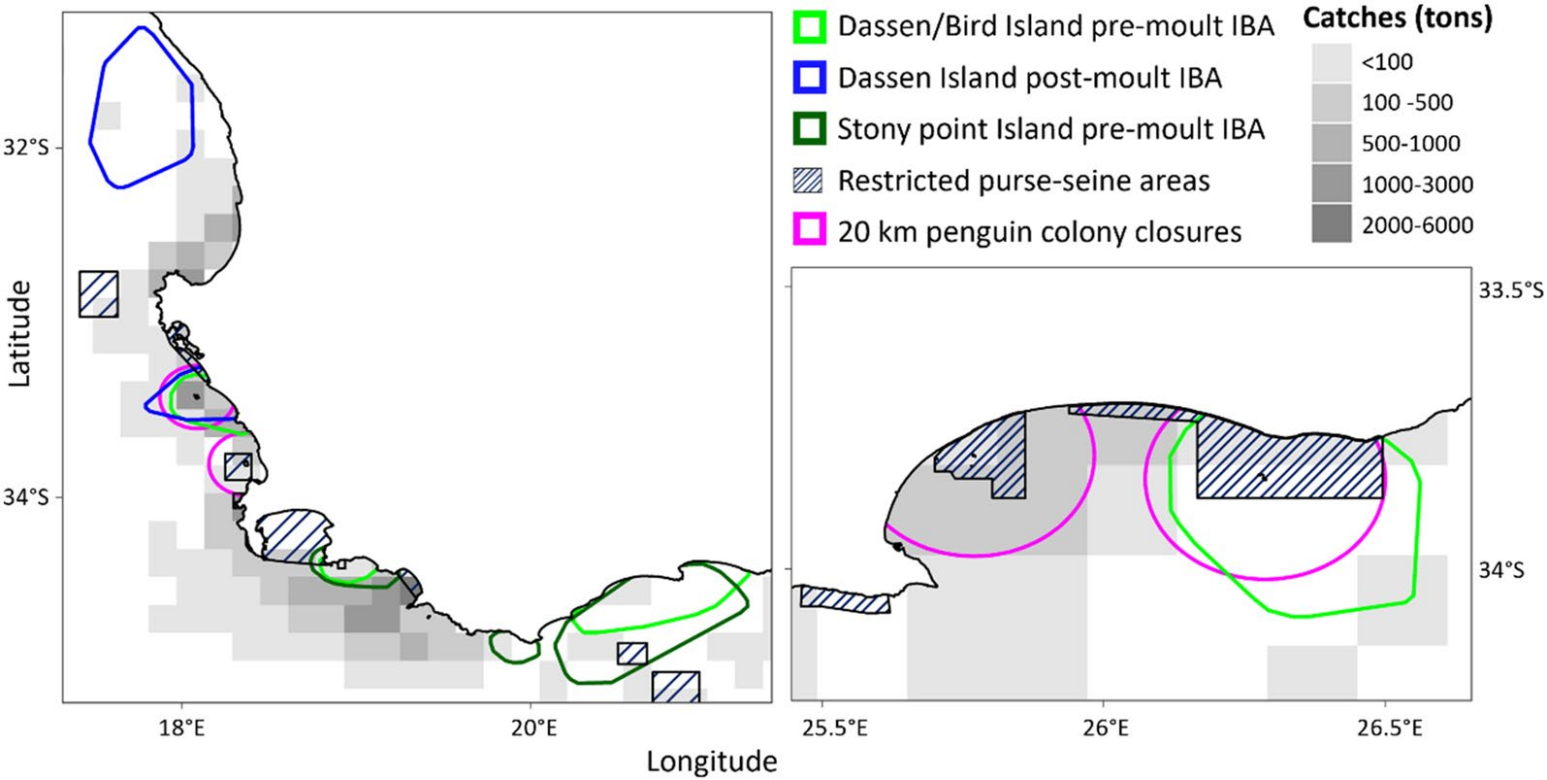
Proposed marine Important Bird Areas of pre- and post-moult African penguins from (**a**) Dassen Island and Stony Point and (**b**) pre-moult African penguins from Bird Island. Areas that are currently restricted to purse-seine fisheries are indicated, as well as the proposed 20 km purse-seine no-take areas around penguin colonies (Dassen, Robben, Bird and St Croix islands). Shaded areas indicate averaged accumulated catch of sardine and anchovy over 2012–2019 during September to December. Marine Important Bird Areas were only calculated for those colonies and stages that had > 10 individuals tracked.

All three pre-moult IBAs and the Dassen Island post-moult IBA (Fig. 4) had representativeness scores > 85% (Table 3). Existing no-take zones for purse-seine fisheries, i.e. within MPAs, had little overlap with these IBAs, except for the Bird Island pre-moult IBA where there was a 32% overlap (Table 3). The IBAs overlapped substantially with the proposed 20 km no-take zones around Dassen, Robben, St Croix and Bird islands, but not Stony Point (Table 3). Despite this variable overlap between IBAs and 20 km no-take zones, birds spent a substantial proportion of time within 20 km (16–67%) and 50 km (30–89%) of their colonies during both pre- and post-moult stages (Table 3).

**Table 3:** (a) Representativeness of the Important Bird Areas (IBA) calculated for pre- and post-moult African penguins dispersing from three colonies.

Colony	Bird Island		Dassen Island		Stony Point
Stage	Pre-moult	Post-moult^a^	Pre-moult	Post-moult	Pre-moult
(a) Representativeness of data (%)	99.9		94.1	94.9	85
**(b) Percentage of IBA overlapping with**					
Existing no-take zones	31.8		8.7	0.7	9.7
Existing no-take zones and proposed closures	70		29.2	17.2	9.7
**(c) Percentage of time spent with**					
20 km excluding land fixes	67.0 ± 22.1	39.9 ± 20.9	15.5 ± 16.4	48.5 ± 36.9	23.6 ± 14.0
20 km including land fixes	75.4 ± 13.6	55.0 ± 8.4	27.9 ± 22.4	60.3 ± 33.7	38.0 ± 15.7
50 km excluding land fixes	85.9 ± 14	69.4 ± 13.8	29.5 ± 29.5	60.2 ± 36.0	30.8 ± 17.6
50 km including land fixes	89.0 ± 11.6	77.5 ± 5.7	37.7 ± 28.7	67.6 ± 33.9	43.6 ± 17.9

The percentage of spatial overlap between these IBAs with (b) existing no-take zones to purse-seine fisheries and 20 km radius around the four experimental no-take zones islands (Dassen, Robben, St Croix and Bird islands). (c) The percentage of time spent at-sea spent within 20 km and 50 km of dispersal colonies, including and excluding points on land.

^a^IBA not estimated for data groups with fewer than 10 individuals.

## Discussion

Successful conservation management of seabirds’ marine habitat should incorporate all life history stages of the seabirds. We show that although pre- and post-moult adult African penguins have much larger distributions compared to during the breeding season and disparate distributions during the pre- and post-moult stages, they use waters within 20 and 50 km of their colonies intensively. This suggests that the 20 km no-take zones around colonies, proposed to alleviate competition for resources between breeding African penguins and fisheries, will also benefit the penguins outside of the breeding season. Our research highlights the need for stage-specific conservation and management of mobile species.

The pre- and post-moult adult African penguins travelled up to 600 km away from their colonies, greatly exceeding the maximum foraging range of breeding penguins(< 50 km)^41,56^. Similarly to the initial dispersal of juvenile African penguins^43^, the pre-and post-moult adults rarely exceeded the 200 m depth contour but did not range as far as the juveniles. Sherley et al.^43^ showed that juvenile African penguins from Dassen Island went northward of their colony to approximately 20°S whereas all pre- and post-moult Dassen Island adults in this study remained below 29°S. The maximum extent travelled along the west coast was similar for pre-moult adults and juvenile penguins dispersing from Stony Point (< 30°S). However, whereas the core areas for the Stony Point juvenile penguins was along the west coast^43^, the core area of the Stony Point pre-moult penguins was along the south coast, east of Cape Agulhas. Together with the findings of Sherley et al.^43^, we have shown that areas north of St Helena Bay on the west coast, in the vicinity of the Stony Point colony and east of Cape Agulhas are important to penguins outside of the breeding season (i.e. the pre- and post-moulting and juvenile stages) and should be adequately reflected in Marine Spatial Planning initiatives within South Africa.

Travelling to distant foraging areas during the pre- and post-moult adults^19,30,63–73^ and juveniles^74–76^ stages is known for other penguin species. Due to the aggregation of high densities of breeding seabirds foraging from a central location, prey availability is thought to decline outward from these central locations as the breeding season progresses, likely leading to increased competition for available prey^77,78^. These large migrations to distant foraging habitats by the African penguin outside of the breeding season may be a relic of this mechanism, wherein the penguins disperse to distant and likely more profitable foraging areas once they are no longer responsible for dependant young (i.e. they are no longer constrained around a central location).

The pre-moult distribution of African penguins departing from Dassen Island and Bird Island showed significant inter-annual variability. Plastic foraging distributions during the pre- and post-moult stages has been demonstrated for several penguin species^19,63,64,68^ and other marine predators such as volant seabirds and seals^79–84^. The marine environment is highly dynamic with numerous biophysical factor determining the spatio-temporal distribution of prey^85^. Behavioural plasticity, such as plastic distributions in seabirds, may be an adaptive trait to locate ephemeral prey^86^. This is likely the case for pre-moult African penguins as plasticity in foraging behaviour as a response to variable prey distribution has been demonstrated for this species during the breeding season^87,88^.

Pre-moult African penguins in the Western Cape (Dassen Island and Stony Point) travelled significantly further than those in the Eastern Cape (Bird Island). Most of the Western Cape pre-moult penguins travelled south-eastward along the South African coastline and had core distributional ranges east of Cape Agulhas. The south-eastward movement of these birds mimic the relatively recent south-eastward shift in distribution of their main prey: anchovy and sardine, again highlighting the possible plastic foraging distribution of these birds during this stage. Due to compounding effects of environmental changes and intensive fishing pressure along the west coast, the distribution of the two forage fish species have shifted south-eastward, east of Cape Agulhas, with sharp decreases in sardine abundances along the west coast^89–91^. The significantly greater distances travelled and areas covered by Dassen Island pre-moult penguins compared to those from the others colonies indicates that this may be as a result of shifting prey distributions and as a consequence they expend the most energy to fatten up during this crucial life history stage, compared to birds at other colonies. This may compromise their ability to accumulate sufficient body reserves for their annual moult, which may influence post-moult survival or recovery, and subsequently, pre-breeding condition and breeding success. The Dassen Island population has shown one of the fastest rates of decrease since 1999^47^ and the survival of African penguins from west coast colonies has been linked to regional estimates of spawner biomass of sardine in the preceding non-breeding season^23,39,44^. Our results provide support for these findings and highlight the importance of prey availability to these birds during the pre- and post-moult life-history stages.

African penguins from Bird and Dassen islands used different areas during their post-moult trips compared to pre-moult trips. However, the distribution of post-moult birds from both colonies overlapped with areas of upwelling and associated cool sea surface temperatures and high chlorophyll-a concentrations. The core ranges of post-moult penguins from Bird Island extended north of Port Alfred, along the eastern edge of the Agulhas shelf where regular upwelling is associated with relatively high prey biomass^92^. Post-moult penguins from Dassen Island mostly travelled to an area north of St Helena Bay, which prior to the south-eastward shift in the distribution of anchovy and sardine, was an important spawning area for sardine^89,93,94^. During summer, when the post-moult penguins were tracked, there is an increase in wind-driven upwelling at both of the sites which promotes phyto- and zooplankton productivity^95–99^, both important prey items of sardine and anchovy^100,101^. African penguins are attracted by olfactory cues, i.e. dimethyl sulphide that is released by phytoplankton^102^. Higher concentrations of such olfactory cues during summer, both within the St Helena Bay and Port Alfred areas, may explain the attraction of penguins to these areas during the post-moult stage (November/December). Alternatively, the penguins may target other cues that attract them to the cold sea surface temperatures and high chlorophyll-a concentrations typical of upwelling in these regions and also attract Cape gannets *Morus capensis*^42^ and juvenile African penguins^43^. Previous tracking studies of pre-moult^30,63,64,70,71^ and post-moult^19,66–70,72,73^ penguins have shown that many species have strong associations with environmental variables (e.g. chlorophyll-a concentrations) or physical features (e.g., oceanographic fronts) where their prey are concentrated.

Post-moult body condition may also have some bearing on the large disparity in foraging distributions of pre- and post-moult African penguins from Dassen Island. Due to the energy demands of their catastrophic moult, African penguins are in poor body condition at the onset of the post-moult period^32^. This may impede their ability to travel to distant foraging grounds, such as the Agulhas shelf habitat visited during their pre-moult stage, especially if there is limited prey available close to their moulting sites to improve body condition prior to departure to distant foraging areas. It may be that the penguins drift more or less passively to the area north of St Helena Bay within the jet current that flows northward along the continental shelf^98^. However, Sherley et al.^43^ found that juvenile African penguins, moving in the same direction and to similar areas as the Dassen Island post-moult adults, were swimming actively to reach this area. Further fine-scale investigation into drivers of adult penguin movements outside of the breeding season is needed.

Marine Important Bird Areas for pre-moult African penguins from three of their six largest colonies^47^, and post-moult African penguins from Dassen Island were identified. All four of these newly identified areas were highly representative of their colonies’ populations (as determined by their representativeness scores, see “Methods” for more details). A small proportion of these IBAs overlap with existing no-take zones for purse-seine fisheries. However, large proportions of the Dassen Island and Bird Island IBAs overlapped with the proposed 20 km no-take zones around four major colonies^103^. Positive effects of these 20 km no-take zones have been demonstrated for foraging and breeding parameters of breeding African Penguins^24,52,53^; ours is the first direct evidence of possible positive impacts on other life-history stages. Several studies have shown strong correlations between the foraging performance of seabirds and foraging conditions during the non-breeding season^25,63,104^ and the consequences of this on seabird species population trajectories^21,105–107^. We show that the waters within 20 km and 50 km of an African penguin’s colony are used intensively by pre- and post-moult penguins. As the breeding season of the African penguin is protracted and asynchronous with moulting and breeding individuals being present in the colonies throughout most of the year^108–110^, improved foraging conditions close to their colonies could have appreciable benefits for African penguins year-round. Given the endangered status of the African penguin, linked to their rapidly declining population^47^, these areas should receive high conservation priority. However, the much more extensive distribution of pre- and post-moult and juvenile^95^ African penguins compared to that of breeding penguins highlights the need for life history stage-specific spatial management and conservation strategies^10–12^.

## Conclusions

The congruent population declines of the African penguin, Cape gannet and Cape cormorant *Phalacrocorax capensis* in South Africa have been attributed to insufficient availability of high quality prey or increased foraging effort to reach profitable foraging areas, resulting in lower reproductive success and survival^40–44^. A key strategy to conserve these endangered seabirds should be to increase opportunities for the birds to access these highly mobile prey while they are available in important foraging areas during all life history stages. No-take zones around major colonies have been proposed as a strategy to improve African penguin breeding success^24,52,53^. Our findings suggest that such fishing closures may also improve prey availability to penguins outside of the breeding season, potentially improving their survival and breeding propensity. However, future research should investigate oceanographic drivers of the penguins’ pre- and post-moult distribution as this will help disentangle the differential foraging distributions between important life-history stages and elucidate the relative influences of natural versus anthropogenic drivers of prey availability for these birds. Marine spatial planning, in the form of MPAs or no-take zones, has been identified as one of the most appropriate tools to manage prey depletion by fisheries^5–8^ and these measures should be expanded to incorporate important foraging areas of African penguins outside of their breeding season, such as the pre- and post-moult stages and the initial dispersal of juvenile birds.

## Methods

### Data collection

The lifecycle of the African penguin is relatively asynchronous and protracted compared to other penguin species as breeding and moulting penguins are often present in the colonies year-round^109,110^. However, in general, breeding peaks during the winter months (February–September and January–July at colonies to the west and east of Cape Agulhas, respectively^108–110^) with the majority of birds moulting during early summer from September to January^108–110^. Using re-sightings of flipper bands, the pre- and post-moult stages of African penguins have been estimated to be c. 35 and c. 42 days in length^109,111^, with birds either returning to their breeding colonies or colonies closer to their pre-moult foraging grounds to moult^112^. The land-based moult is c. 21 days^32^**.**

Between 2012 and 2019, three types of tracking devices were deployed on African penguins before and after their annual moult (KiwiSat202, SirTrack, 58 × 28 × 18 mm with 180 mm antennae, 40 g; CatLog-S, Perthold Engineering LLC USA, 50 × 22 × 8 mm, 34 g and GPS-GSM nanoFix®GEO, Pathtrack Limited, 63 × 20 × 18 mm with 40 mm antennae, 25 g), hereafter referred to as ‘pre-moult’ and ‘post-moult’ stages, at Dassen Island (33°25′S, 18° 05′E, 2012–2019) and Bird Island (33° 50′S, 26° 17′E; 2012–2015) and Stony Point (34° 22′S, 18° 53′E, 2018–2019; Table 1). From August to October, loggers were deployed on pre-moult breeding adults identified as those with late stage chick(s) ready to fledge (i.e. chicks with few to no downy feathers) ^109^. During November, loggers were deployed on adults in the last stages of their feather moult (i.e. few to no old feathers). Loggers were attached to the feathers on the dorsal midline of the bird’s lower back using tesa^®^ tape (Beiersdorf AG, Germany) and secured with cable ties and cyanoacrylate glue (Loctite 401^®^). Due to the lower battery life of the PTT and Catlog GPS devices used between 2012 and 2015 (Table 1), these devices were scheduled to only record locations at night, between 18:00 and 24:00 GMT and 21:00 and 22:00 GMT, respectively, to maximise the devices’ ability to log a position while the penguins rested on the surface during night-time hours. The greater battery life of the solar-powered Pathtrack GSM-GPS devices, used between 2017 and 2019, allowed for locations of the penguins to be recorded every hour.

All methods were approved by South African National Parks (permit number: MOSEC1122), Cape Nature (permit numbers: 0056-AAA007-00087; 0056-AAA007-00033, 0056-AAA007-00171, CN44-87-17102) and the South African Department of Forestry, Fisheries and the Environmental (RES2012/78, RES20132/77, RES2014/49, RES2015/46, RES2016/38, RES2017/42, RES2018/51, RES2019/17) This project received ethics clearance from University of Cape Town’s Science Animal Ethics Committee (2012/V47/PR and 2015/V12/PR) and BirdLife South Africa’s Animal Ethics Committee (2018/01/B). Methods were performed in accordance with the relevant permits and regulations. The study was carried out in compliance with the ARRIVE Guidelines.

### Pre- and post-moult distribution of African penguins

There was considerable variability in the number of days (defined as the full 24 h of each calendar date) individuals were tracked (range 2–78 days). Number of days tracked were defined from the first location recorded at sea to either the last location recorded at sea before evidence of moulting was noted (> 48 h on land) or the last transmitted location at sea. To ensure that tracks were representative of pre- and post-moult foraging trips, only tracks > 20 days were analysed. This is because it was found that for penguins which moulting could be determined, all pre-moult foraging trips were greater than 20 days (range 21–57 days; Table 1) Penguins roosted on land regularly (both at the deployment colony and at other colonies), therefore, locations on land were removed and tracks were split into trips between these land-based events. Tracking data were filtered for erroneous fixes based on transit speeds of greater than 12.4 km·h^−1^^113^ using the R package *argosfilter*^114^.

For each tracked bird, the following path metrics were calculated: (1) the maximum distance travelled away from the deployment colony, (2) the path length from the deployment colony to the maximum distance using the sum of great circle distances between consecutive locations and (3) the area covered by their core range (further referred to as ‘individual core area’). These individual core areas were estimated as utilisation distributions ^UD^^115^ using the R package *adehabitatHR*^116^ with a smoothing factor (h) of 7 km following Dias et al.^58^. The isopleth that demarcated each individual core area was estimated based on optimal isopleth value selection (OIVS), following Vander Wal and Rogers^117^. The OIVS method uses the exponential relationship between the proportion of home range area used by an individual and the isopleth volume to identify thresholds (slope = 1) delineating areas of maximum use. The OIVS was applied to each individual and the mean optimal isopleth value of all individuals (54% UD) was taken to represent the core range of the birds and used in all individual core area estimations.

Due to different sampling regimes between 2012 and 2015 (locations only recorded at night) and 2017 and 2019 (1-h intervals), all tracks were down-sampled and linearly interpolated to have one location per day prior to the calculation of path metrics. Using data collected between 2017 and 2019, we investigated the impact of down-sampling the tracks by testing for significant differences in path metrics calculated with the original (24 locations per day) and down sampled data (1 location per day) using a permutational analysis of variance test (perANOVA, 5000 permutations). Maximum distance (p = 0.87) and individual core area (p = 0.42) were found to be similar between the sampling regimes. However, path length (p < 0.01) was significantly longer when sampled more frequently. Despite this bias, we retained path length because it was highly correlated between the two sampling regimes (Spearman’s correlation coefficient = 0.93). In addition, the influence of the type of tracking device on path metrics and number of days tracked was investigated using a perANOVA. Due to the relatively low sample of different devices used for different stages and colonies (Supplementary Fig. S1 online), comparisons were limited to Dassen Island’s pre-moult stage. Maximum distance (p = 0.43), individual core area (p = 0.14) and path length (p = 0.26) were found to be similar between device types however number of days tracked was significantly greater for birds equipped with PTTs compared to birds equipped with GPS-GSMs and Catlogs. However, the majority of the pre-moult trips tracked with GPS-GSMs were complete even though the number of tracked days by these devices were lower than that of PTTs and Catlogs (Table 1, trips were labelled as complete if there was evidence of the bird remaining on land for > 48 h before data stopped being transmitted and could thus be assumed to be moulting). Data from all tracking devices were therefore retained. Whether the birds were moulting could not be determined for those tracked by PTTs and Catlogs as these devices were only set to record at night which prevented us from distinguishing whether birds were roosting on land overnight or moulting. Path metrics appeared to be greater for post-moult Dassen Island birds that were tracked with PTTs compared to those tracked with GPS-GSMs (Supplementary Fig. S1 online). These data were still pooled and used in further analyses as between year comparison were not made.

Permutational analysis of variance tests (perANOVA, 5000 permutations) were also used to assess path metrics differences among colonies during the pre-moult stage. Due to the lack of post-moult data from Stony Point, differences were not investigated among colonies during the post-moult stage. Differences between pre- and post-moult path metrics were tested separately for Dassen Island and Bird Island. Dunn’s post-hoc tests with Bonferroni corrections for multiple comparisons were applied to all significant permutation test results (package: dunn.test)^118^.

Individual penguin data were then pooled per stage and year (hereafter referred to as data group) to investigate per-stage and annual differences in core (54% UD) and distributional (90% UD) ranges. Comparisons were limited to data groups that had five or more individuals (Table 1). Overlap between data group UDs was calculated using Bhattacharyya’s affinity^119^. The null hypothesis of no spatial difference in range use was tested by permuting the data group labels (year or stage) 1000 times and calculating overlap for each permutation (e.g. stage or year, as appropriate). The p-values for the permutation tests were estimated as the proportion of times the observed overlap was greater than the permuted overlap. Following the same procedure, the influence of down-sampling the data to one location per day was tested to quantify overlap between UDs calculated with the original (24 locations per day) and down-sampled data (one location per day) between 2017 and 2019 for pre-moult penguins from Dassen Island. No significant differences were found (54% UD: proportional overlap = 0.89; p-value = 1.00) and 90% UD: proportional overlap = 0.94; p-value = 1.00).

To visualise purse-seine fishing pressure within the inshore regions of South Africa’s Exclusive Economic Zone, sardine and anchovy catches caught between September and December were aggregated into a grid with a 0.16° resolution for each year of the study (2012–2019, except for 2016 due to incomplete data). An average was then taken across years to represent the relative purse-seine fishing pressure. Locations, dates and tonnage of anchovy and sardine hauls (inclusive of targeted catch and bycatch) from 2012 to 2019 were taken from vessel logbooks (Department of Forestry, Fisheries and the Environment unpub. data).

### Identification of marine Important Bird Areas

Following methods developed by Birdlife International^58,59^, IBAs for the African penguin during their pre- and post-moult stages were identified using the R package *track2KBA*^120^. An IBA is defined as a site that is known to regularly hold significant numbers of globally threatened species or a site that supports > 1% of the global population of a congregatory bird^59,121^. IBAs were identified for each colony and stage (data groups) but only if a data group consisted of > 10 individuals (Table 1). For each data group, areas where > 10% of individuals’ core areas overlapped were identified. These areas were then assessed to check if they were representative of the tracked population and thus adequately described the at-sea distribution of the data group by calculating the ‘representativeness’ of the data group (function: *repAssess,* R package *track2KBA*^120^). They were deemed representative if their representativeness score was > 70%. A > 70% ‘representativeness’ score of the data allows for the assumption that the sampled tracks were able to adequately identify commonly used or important areas of the population. It does not however account for the different duration of the tracks and associated missing fixes that may result from random loss of transmission due to factors like depleted battery or device malfunction.

To enhance the practicability of management zones, spatial polygons were aggregated to minimise the boundary-to-area ratio, following the methods of Handley et al.^122^. Specifically, any isolated polygon or hole within a larger polygon, smaller than 5% of the total area identified, was removed or filled, respectively, using the R package *smoothr*^123^. Polygons were further merged if the great circle distance between their centroids was < 5% of the distance between the two most distant polygon centroids. The final boundaries of sites identified for each data group were delimited by a minimum convex polygon.

The relative overlap of IBAs with existing no-take zones for purse-seine fisheries^124^ was calculated. Following this, the relative overlap of IBAs with the proposed 20 km no-take zones to purse-seine fisheries around Dassen, Robben, Bird and St Croix islands was calculated. To investigate the importance of these areas to pre- and post-moulting African penguins the percentage of time spent within 20 km (representative of the proposed no-take zones) and 50 km (representative of the maximum foraging range of breeding penguins)^41,56^ of a penguin’s colony was calculated (using the percentage of fixes). Percentages were calculated both excluding and including locational fixes recorded at the colony.

Values are given as means ± standard deviations, unless otherwise specified. Significance is set at p ≤ 0.05. All data analyses were performed in the R statistical environment R version 4.0.5.^125^.

## Supplementary Information

Supplementary Information.

## Data Availability

Requests for tracking data used in this study may be made via the BirdLife International Seabird Tracking Database (http://www.seabirdtracking.org).
