# Supplementary material for: Important marine areas for endangered African penguins before and after the crucial stage of moulting
Source: Sci Rep. 2022 Jun 8;12:9489. doi: 10.1038/s41598-022-12969-w (PMC9177839; doi:10.1038/s41598-022-12969-w)
Supplement: Supplementary file 1 — Supplementary Information. [file 41598_2022_12969_MOESM1_ESM.docx]

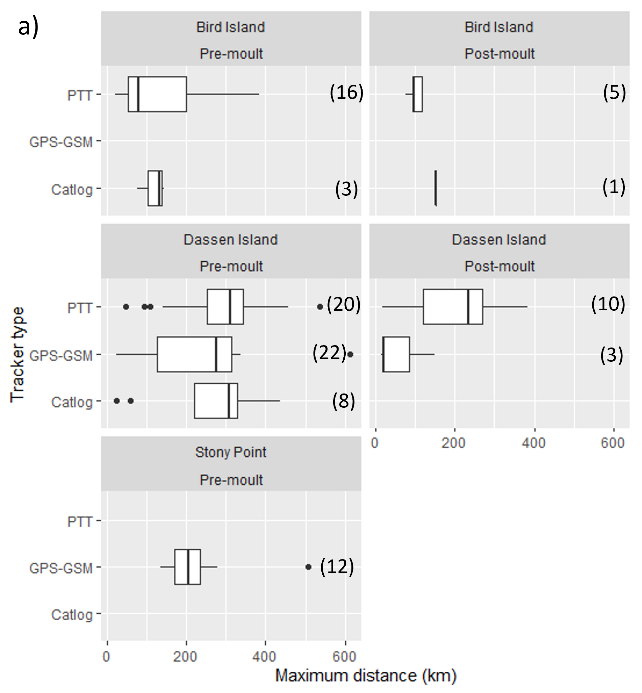

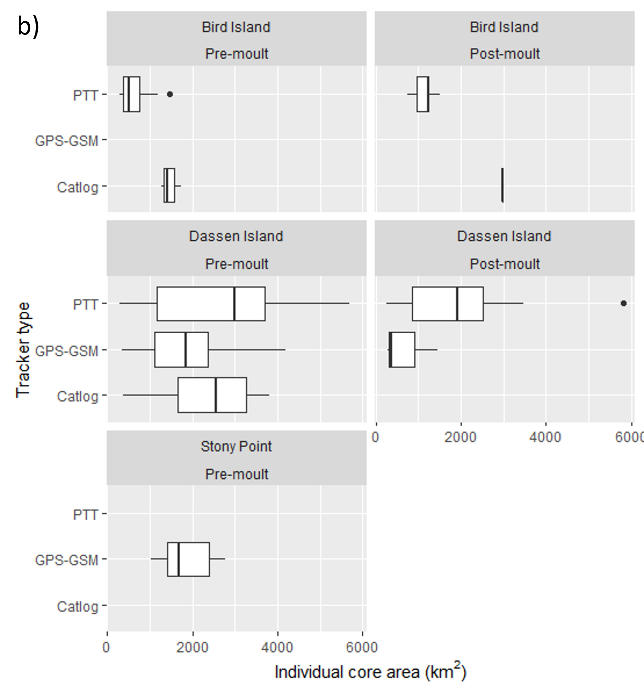

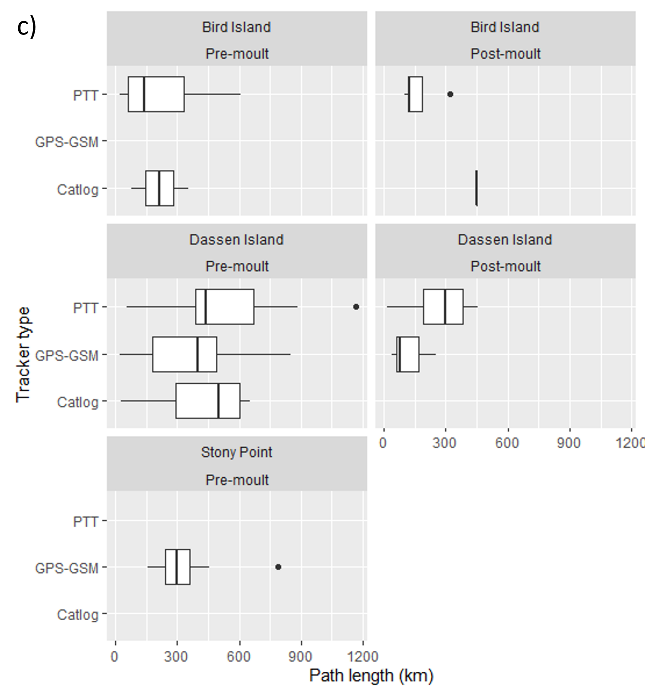

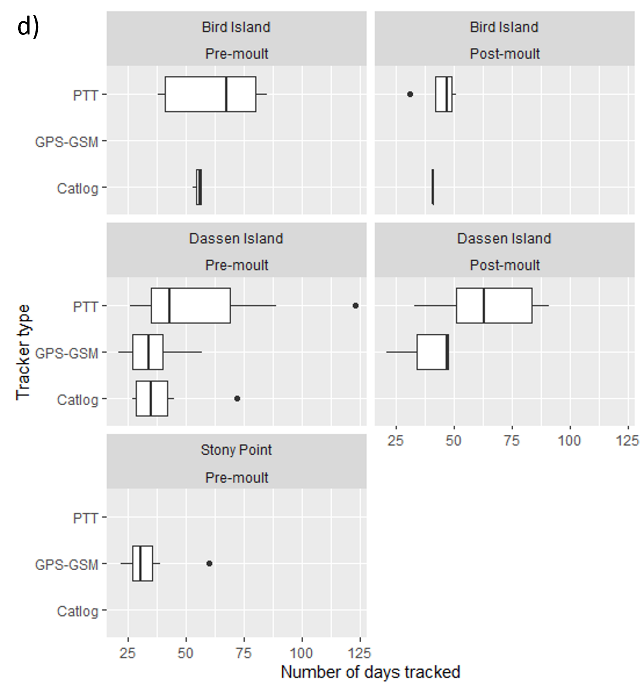


**Supplementary Fig S1.** Box plots (median, interquartile (IQR_3_ and IQR_4_) range, minimum (IQR_3_*1.5) and maximum (IQR_4_) values and outliers) and sample size indicated in brackets of non-breeding African penguin path metrics during pre-moult (left) and post-moult (right) stages among different tracking device types used within the study. The path metrics are a) maximum distance travelled from deployment colony b) area of individual core areas (i.e. 54% utilisation distribution - UD), c) path length of maximum distance travelled from deployment colony and d) number of days tracked.

**Supplementary Table 1.** Maximum distance travelled from deployment colony, area of individual core areas (i.e. 54% utilisation distribution and path length to maximum distance travelled from deployment colony (mean ± STD) of non-breeding African penguins during pre-moult and post-moult foraging trips departing from Bird Island, Dassen Island and Stony Point.

| Colony | Stage | Maximum distance (km) | Individual core area (km^2^) | Path length (km) |
| --- | --- | --- | --- | --- |
| Bird Island | Pre-moult | 127.24 ± 100.34 | 762.18 ± 466.27 | 217.52 ± 187.77 |
|  | Post-moult | 108.89 ± 26.48 | 1445.48 ± 789.84 | 215.67 ±139.19 |
| Dassen Island | Pre-moult | 261.10 ± 132.16 | 2217.56 ± 1305.16 | 425.89 ± 244.78 |
|  | Post-moult | 171.79 ± 126.66 | 1760.80 ± 1610.77 | 234.16 ± 150.90 |
| Stony Point | Pre-moult | 224.10 ± 98.33 | 1850.07 ± 576.63 | 334.36 ± 16877 |
